# SpineCor treatment – the Spanish experience. First results

**DOI:** 10.1186/1748-7161-7-S1-O39

**Published:** 2012-01-27

**Authors:** C Herrero, E Herrero

**Affiliations:** 1 Centro de Traumatologia y Ortopedia Pediatrica y del Adolescente (CTPA), Girona, Spain

## Purpose of the study

The purpose of this study was to evaluate the effectiveness of the Dynamic SpineCor brace as a new treatment for adolescent idiopathic scoliosis [1,2].

## Materials and methods

117 scoliotic patients at our clinic accepted the SpineCor brace and 34 (30 females and 4 males) have already finished the treatment. Assessment of brace effectiveness included percentage of patients who have 5° or less curve progression and the percentage of patients who have 6° or more progression at skeletal maturity. We employed the SRS22 and CAVIDRA questionnaire for the evaluation of patients’ quality of life while using the SpineCor System.

## Results

Success of the treatment (stabilisation or correction) was achieved in 88.3% of patients and only 11.7% had a progression of their Cobb angle. Out of 34 patients, 18 (52.9%) had a correction and 12 (35.3%) had a stabilisation of their initial Cobb.

## Conclusions

The SpineCor brace is an effective treatment for the adolescent idiopathic scoliosis. Even though until now we have a small number of patients treated, the results are extremely promising.

## References

[B1] Coillard C Circo AB Rivard CH SpineCor treatment for Juvenile Idiopathic Scoliosis: SOSORT award 2010 winner Scoliosis 2010 5 25 10.1186/1748-7161-5-25 21067608 PMC2989306

[B2] Coillard C Vachon V Circo A Beausejour M Rivard CH Effectiveness of the SpineCor brace based on the new standardized criteria proposed by the Scoliosis Research Society for Adolescent Idiopathic Scoliosis J Pediatr Orthop 2007 27 375 379 10.1097/01.bpb.0000271330.64234.db 17513955

